# *Helicobacter pylori* culture positivity and antimicrobial susceptibility profiles (Vancouver, Canada)

**DOI:** 10.1093/jac/dkaf114

**Published:** 2025-04-09

**Authors:** Hasan Hamze, Michael Payne, Aleksandra Stefanovic, Christopher F Lowe, Marc G Romney, Nancy Matic

**Affiliations:** Pathology and Laboratory Medicine, University of British Columbia, 2211 Wesbrook Mall, Vancouver, Bc V6T 1Z7, Canada; Pathology and Laboratory Medicine, University of British Columbia, 2211 Wesbrook Mall, Vancouver, Bc V6T 1Z7, Canada; Department of Pathology and Laboratory Medicine, St. Paul’s Hospital, Providence Health Care, 1081 Burrard St., Vancouver, Bc V6Z 1Y6, Canada; Pathology and Laboratory Medicine, University of British Columbia, 2211 Wesbrook Mall, Vancouver, Bc V6T 1Z7, Canada; Department of Pathology and Laboratory Medicine, St. Paul’s Hospital, Providence Health Care, 1081 Burrard St., Vancouver, Bc V6Z 1Y6, Canada; Pathology and Laboratory Medicine, University of British Columbia, 2211 Wesbrook Mall, Vancouver, Bc V6T 1Z7, Canada; Department of Pathology and Laboratory Medicine, St. Paul’s Hospital, Providence Health Care, 1081 Burrard St., Vancouver, Bc V6Z 1Y6, Canada; Pathology and Laboratory Medicine, University of British Columbia, 2211 Wesbrook Mall, Vancouver, Bc V6T 1Z7, Canada; Department of Pathology and Laboratory Medicine, St. Paul’s Hospital, Providence Health Care, 1081 Burrard St., Vancouver, Bc V6Z 1Y6, Canada; Pathology and Laboratory Medicine, University of British Columbia, 2211 Wesbrook Mall, Vancouver, Bc V6T 1Z7, Canada; Department of Pathology and Laboratory Medicine, St. Paul’s Hospital, Providence Health Care, 1081 Burrard St., Vancouver, Bc V6Z 1Y6, Canada

## Abstract

**Introduction:**

*Helicobacter pylori* is associated with gastrointestinal diseases including gastritis and peptic ulcers. Despite its significance, there is a scarcity of antimicrobial susceptibility testing (AST) data available for this organism in North America.

**Objectives:**

The aim of this study was to assess the AST profile and identify factors associated with *H. pylori* culture positivity in a cohort of patients with refractory *H. pylori* undergoing gastric biopsies.

**Methods:**

We retrospectively reviewed gastric biopsy specimens received for culture between July 2009 and February 2023. We analyzed specimen transport time, Gram smear results, direct urease test findings, culture positivity and AST profiles. Using gradient strip methodology and European Committee on Antimicrobial Susceptibility Testing breakpoints, AST was conducted for amoxicillin, clarithromycin, metronidazole, levofloxacin and tetracycline.

**Results:**

Of 579 biopsy samples received for *H. pylori* culture, 228 (39.4%) tested positive. Samples transported within <1 h had significantly higher odds (1.81 times, *P *< 0.015) of being culture positive compared to those with longer transport times. Smear-positive samples had substantially higher odds (18.8 times, *P *< 0.001) of culture positivity compared to smear-negative. Urease-positive samples demonstrated notably higher odds (7.7 times, *P *< 0.001) of culture positivity compared to urease-negative samples. The collection of isolates from gastric biopsies showed susceptibility rates of 97.3% to amoxicillin, 99.1% to tetracycline, 50.4% to levofloxacin, 25.9% to metronidazole and 12.9% to clarithromycin.

**Conclusions:**

Short sample transport time was associated with improved *H. pylori* recovery rates. In this cohort of refractory *H. pylori* cases, susceptibility rates were high for amoxicillin and tetracycline and low for clarithromycin, metronidazole and levofloxacin. Susceptibility rates remained stable over time.

## Introduction

*Helicobacter pylori* is a fastidious, microaerophilic, urease-positive Gram-negative bacillus.^1^ It is a cause of chronic inflammation of the stomach's mucosal lining.^2^ Its population prevalence is approximately 50% globally and between 20% and 30% in Canada.^1,3^ In low-middle-income countries, *H. pylori* prevalence can be as high as 80%.^4^  *H. pylori* colonization occurs in early childhood and can persist throughout life without effective treatment, with up to 20% of those infected developing gastric or duodenal ulcers. Up to 2% are at risk of developing gastric cancer.^2^ Development and severity of the disease may depend on host, environmental and pathogen factors (e.g. diet, crowding, smoking, poor sanitation and virulence of the strain).^2,5,6^

The management of *H. pylori* infection plays an important role in reducing complications from infection. Treatment regimens vary: some consensus guidelines recommend first-line quadruple therapy with two antibiotics (metronidazole and tetracycline), bismuth subsalicylate and a proton pump inhibitor (PPI) to suppress gastric acid secretion; or three antibiotics (amoxicillin, metronidazole and clarithromycin) and a PPI.^7^ The previously recommended regimen containing a PPI combined with clarithromycin and amoxicillin or metronidazole, and one of the currently recommended quadruple therapy regimens containing metronidazole and tetracycline, both had eradication rates >90% in 2000, but this had dropped to 70% by 2010.^8,9^ Indeed, there is an alarming rise in *H. pylori* antibiotic resistance globally.^10^ There is a need for more effective and tailored antibiotic treatments based on regional antimicrobial susceptibility profiles.

There are limited antimicrobial susceptibility testing (AST) data for *H. pylori* as it is not routinely cultured, given its fastidious nature. Recent surveillance studies were conducted in the UK, Japan and Vietnam demonstrating increasing resistance to clarithromycin and fluoroquinolones, with the last comprehensive global review in 2017.^5,11–14^ Limited AST data for North American *H. pylori* isolates have been published recently, with several regional studies prior to 2017 within the USA and Canada.^3,6,15–23^ Most of these studies do not describe factors affecting laboratory performance or *H. pylori* culture yield. Therefore, the progression of *H. pylori* antimicrobial resistance and the opportunities for laboratories to optimize testing are incompletely understood.

At our centre, patients are first diagnosed with *H. pylori* infection using serology, followed by urea breath testing or stool antigen in some cases. Patients failing empiric antibiotic therapy may undergo gastric biopsies at the discretion of the treating gastroenterologist, which can be submitted for microaerophilic culture and AST. The aim of our study was to determine the AST profile and identify factors associated with successful isolation of *H. pylori* in culture in a cohort of patients with refractory *H. pylori* undergoing gastric biopsies, referred to our center in British Columbia (BC), Canada.

## Methods

We retrospectively reviewed gastric biopsies from refractory cases collected between July 2009 and February 2023 at various hospitals across Bc, Canada, and submitted to the St. Paul’s Hospital microbiology laboratory for *H. pylori* culture and AST. Refractory cases were defined as those failing first-line antibiotic therapy. Gastric biopsies were collected at the discretion of the endoscopist. One gastric biopsy per patient was placed in a sterile screw-top container with a few drops of sterile isotonic saline before transport to the microbiology laboratory. Laboratory data collection included transportation time, Gram smear, direct urease positivity, culture result and phenotypic AST results for amoxicillin, clarithromycin, levofloxacin, metronidazole and tetracycline. Transportation time was defined as the difference in time between sample collection and sample receipt in the laboratory, in hours. A subanalysis of culture yield was performed on gastric biopsies defined to be ‘gold standard positives’ based on a composite reference standard [gastric biopsies testing positive by direct urease test and with curved Gram-negative bacilli (CGNB) seen on Gram stain]. Data were analyzed using IBM SPSS Statistics 2023 software.

Each clinical sample had been processed following standard laboratory protocols. Briefly, each tissue was minced with a sterile scalpel and ground with 0.5 mL sterile saline. A Gram smear and acridine orange smear were performed. The suspension was inoculated onto Columbia agar with 5% sheep blood and chocolate agar and incubated at 35°C in a microaerophilic jar for 10 days. A direct urea slant was inoculated and incubated at 35°C. This was examined at 30 min and 4 and 24 h and discarded at 48 h, if negative. Laboratory technologists read plates to identify small, gray and moist colonies. Isolates were confirmed to be *H. pylori* if they were curved gram-negative bacilli testing oxidase, catalase and urease positive.

AST was performed using guidance from the European *Helicobacter* and Microbiota study group.^24^ Colonies from two- to three-day-old pure culture of *H. pylori* were resuspended in Brucella Broth to a McFarland standard of 3 (approximately 1.0 × 10^9^ colony forming units per mL). This suspension was plated onto Brucella agar along with gradient strips (ETEST^®^, bioMérieux, Canada) for amoxicillin, clarithromycin, metronidazole, tetracycline and levofloxacin. Levofloxacin AST was only routinely performed in our laboratory after 2016. Plates were incubated at 35°C for 72 h in microaerophilic conditions. Interpretations were based on European Committee on Antimicrobial Susceptibility Testing breakpoints.^25^

## Results

### Culture and biochemical testing

A total of 579 biopsy samples were received for *H. pylori* culture during the study period, of which 228 (39.4%) were culture positive (Table 1). Gram smears demonstrated CGNB in 57.0% (130/228) of culture-positive samples, but only 6.6% (23/351) of culture-negative samples (*P* < 0.001). Smear-positive samples had 18.8 times the odds (*P* < 0.001) of being culture positive compared to smear-negative samples. Direct urease testing was positive in 63.2% (144/228) of culture-positive samples versus 18.2% (63/347) of culture-negative samples (*P* < 0.001). Urease-positive samples had 7.7 times the odds (*P* < 0.001) of being culture positive compared to urease-negative samples. Of Gram smears with the presence of polymorphonuclear cells (representing inflammation), 131 (57.5%) were culture positive (*P* = 0.40).

**Table 1.** Total number of isolates and characteristics of *H. pylori* culture-positive compared to culture-negative samples

Variable	Culture positive, *N* (%)	Culture negative, *N* (%)	*P*-value
Total isolates	228 (39.4%)	351 (60.6%)	—
Sample transport time^a^	2.7 h	3.6 h	0.04
CGNB on smear	130 (57.0%)^b^	23 (6.6%)^b^	<0.001
Inflammation on smear	131 (57.5%)^b^	214 (61.0%)^b^	0.4
Urease positive	144 (63.2%)^b^	63 (18.2%)^b^	<0.001

^a^Only samples with a valid transport time were included in this calculation (*n* = 366).

^b^The percentage of total culture positive (*n* = 228), or culture negative samples (*n* = 351), respectively.

### Sample transport

**Figure 1.**
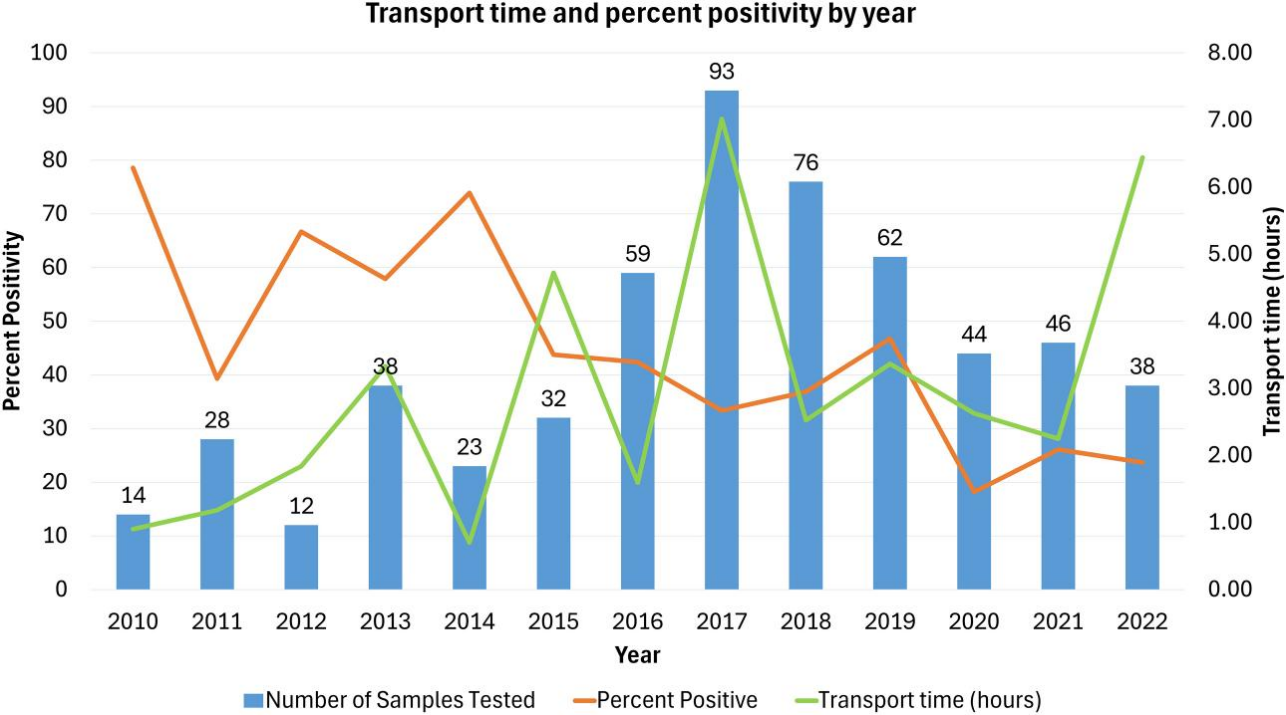
*H. pylori* sample transport time, percent positivity and number of samples by year. Samples collected in 2009 (*n* = 4) and 2023 (*n* = 6) were excluded from this graph due to incomplete annual data.

The average transport time to the laboratory was 2.7 h for *H. pylori* culture-positive samples and 3.6 h for culture-negative samples (*P* = 0.04) (Table 1). Sample transport time and culture percent positivity were inversely proportional (Figure 1). Samples with a transport time of <1 h had 1.81 times the odds (*P* < 0.015) of being culture positive compared to those with a transport time of >1 h. Samples with a transport time greater than 40 h were excluded for the purpose of transport time analysis, as they were considered outliers (*n* = 8).

### Antimicrobial susceptibility testing

**Figure 2.**
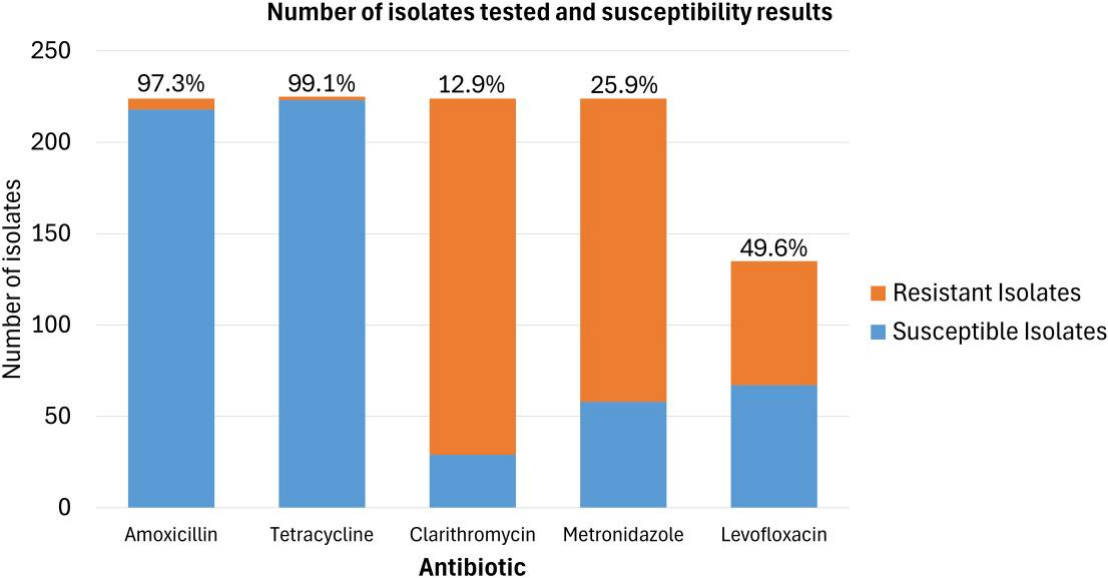
Number of *H. pylori* isolates and susceptibility of antibiotics tested. Percentage value denotes the percentage of isolates that were susceptible.

**Figure 3.**
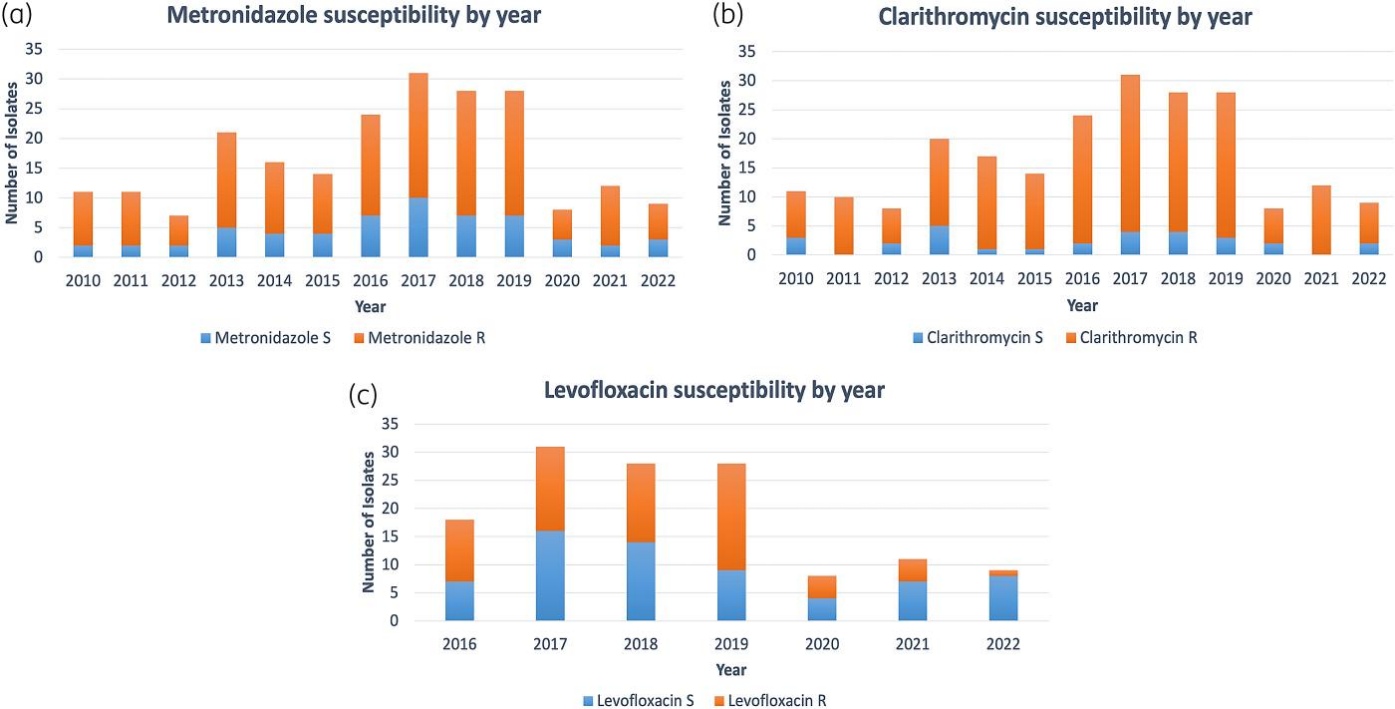
Metronidazole (a), clarithromycin (b) and levofloxacin (c) susceptibility results for *H. pylori* isolates by year. Samples collected in 2009 and 2023 were excluded from these graphs due to incomplete annual data.

*H. pylori* isolates were 99.1% susceptible to tetracycline (223/225), 97.3% to amoxicillin (218/224), 50.4% to levofloxacin (68/135), 25.9% to metronidazole (58/224) and 12.9% to clarithromycin (29/224) (Figure 2). Clarithromycin and metronidazole demonstrated the largest proportion of resistant isolates (361/446), compared to tetracycline and amoxicillin, which demonstrated the smallest proportion of resistant isolates. Susceptibility rates remained mostly stable over time (Figure 3). Only four *H. pylori* isolates were susceptible to all five antibiotics. Levofloxacin susceptibility has been trending upwards from 2020 to 2023, although the number of samples tested was small compared to previous years.

### Composite reference standard subanalysis

Among the gastric biopsies deemed to be ‘gold standard positives’ by the composite reference standard, 95/108 (88%) were culture positive. The average transportation time of culture-positive specimens was similar to those that were culture negative in this subset (2.7 versus 2.0 h, *P = *0.70). Antimicrobial susceptibility rates for isolates in this subset were 98.9% for amoxicillin, 91.6% for tetracycline, 21.1% for metronidazole, 10.5% for clarithromycin and 41.7% for levofloxacin, consistent with the findings of the main analysis.

## Discussion

*H. pylori* isolation methods have largely remained the same in recent history, relying on standard culture.^26^ However, optimal methods of isolation remain to be established. Overall, we found that smear positivity for CGNB and direct urease testing were highly associated with the isolation of *H. pylori* in culture. A few studies have examined the role of Gram stain and direct urease testing for the isolation of *H. pylori*. One study found that Gram stain and direct urease testing was 88% sensitive as a rapid method of detection when one of these tests was positive.^27^ Another demonstrated that 92.8% of culture-positive samples had positive Gram stains when used as a rapid screening method.^28^ In both studies, samples were immediately transported to the microbiology laboratory for analysis after collection, which likely improved positivity rates. Our study did identify several culture-negative biopsies with either CGNB seen on Gram stain (6.6%) or direct urease positivity (18.2%), suggesting that *H. pylori* may have been initially present in the sample but failed to grow in culture. It is also possible some gastric biopsy samples were contaminated with oropharyngeal flora, leading to false-positive direct urease results. Nevertheless, our results demonstrate the value of performing both Gram staining and direct urease testing to increase the sensitivity for *H. pylori* detection.

The effective isolation of *H. pylori* from gastric biopsy specimens is challenging due to the impact of transport conditions, such as sample transport time, temperature and exposure to ambient air.^29^ Published data on this topic are limited, with mixed results. The effect of sample transport time and temperature is somewhat controversial, as some studies have shown improved recovery with rapid transport, and others demonstrated that *H. pylori* can survive at room temperature for 24 h without loss of ability to recover.^30–34^ In a more recent study from China comparing isolation of *H. pylori* based on different transport times, the positive culture rate from the 48-h group was significantly lower than the 24-h group (26.25% versus 33.73%).^35^ Culture positivity rates in our study appear to have decreased over time, while transport time increased, possibly due to increasing demand from more remote healthcare sites outside of our hospital. Our study demonstrates significantly improved isolation of *H. pylori* for samples with a short transport time, particularly less than 1 h. Among the ‘gold standard positive’ samples, transportation time was not significantly different for culture-positive versus culture-negative samples, although the total number of culture-negative samples in this subset was small. For remote hospitals that rely on transport to a centralized laboratory for *H. pylori* testing, rapid transportation in less than 1 h can be a significant challenge. These factors highlight the need for more rapid, reliable methods for the prediction of antimicrobial resistance.

One potential solution to overcome the barrier of transport time and the challenges of phenotypic susceptibility testing is the implementation of newly emerging technologies, such as next-generation sequencing and real-time PCR.^36,37^ Such molecular techniques have enabled the identification of molecular mechanisms implicated in phenotypic resistance to antibiotics in *H. pylori*.^38^ Based on current data, genotypic prediction of resistance to clarithromycin, levofloxacin and amoxicillin has been found to be accurate. However, for metronidazole, tetracycline and rifabutin, genotypic prediction is not always congruent with phenotypic AST.^39^ For metronidazole, its complex mechanism of action makes identifying resistance targets challenging.^40^ For tetracycline and rifabutin, the small number of resistant isolates makes it difficult to obtain an accurate analysis of the genotypic–phenotypic relationship.^41^

Our study is the first to examine cumulative provincial antimicrobial susceptibility in refractory cases. Our susceptibility results demonstrated high resistance rates to clarithromycin (87.1%), metronidazole (74.1%) and levofloxacin (50.4%). One Canada-wide study in 2000, which did not comment on whether cases were refractory, estimated metronidazole resistance ranged from 11% to 48%, while resistance to clarithromycin was up to 12%.^16^ Resistance to levofloxacin in Canada was estimated to be less than 10% in 2004.^19^ In a more recent 2015 study in Northeastern Ontario with a sample size of 20, resistance rates were found to be 40% for clarithromycin, 35% for metronidazole and 30% for levofloxacin.^20^ In the USA, a systematic review from 2011 to 2021 demonstrated a pooled resistance rate of 29.5% for clarithromycin, 38.7% for metronidazole and 34% for levofloxacin.^23^ Elsewhere, the highest rates of resistance to clarithromycin were found in South Asia (27%), East Mediterranean (33%) and West Pacific (34%), which were significantly higher than in Africa (15%) and Southeast Asia (10%).^14^ Similarly, metronidazole resistance was 69% in South Asia, which was much higher than in Europe (32%), while it was highly variable in Southeast Asia, ranging from 26% to 96.4%.^14,42^ Levofloxacin resistance was highest in South Asia (34%).^43^ In these studies, it was not determined whether samples were taken from treatment-naive patients, or those with refractory disease. It is important to note that our study examined endoscopy samples that are typically obtained for patients who are refractory to first-line therapy, and as such, resistance rates are expected to be higher. One European study determined antibiotic resistance rates in refractory patients were 66% for clarithromycin, 54% for metronidazole and 28% for levofloxacin, which are more consistent with our findings.^44^

Our susceptibility results demonstrate low rates of resistance to amoxicillin (2.7%) and tetracycline (0.9%). In Canada, the prevalence of amoxicillin resistance was estimated to be <2% based on a study in 1997.^17^ In the same Northeastern Ontario study referenced earlier, none of the isolates were resistant to amoxicillin or tetracycline (*n* = 20).^20^ Our findings are consistent with previous data demonstrating low rates of resistance to amoxicillin and tetracycline in Canada. As such, these antibiotics are currently effective options for *H. pylori* eradication. In other areas of the world, however, amoxicillin resistance was high: Africa (38%), South Asia (23%) and the East Mediterranean (14%).^14^ Tetracycline resistance was 16% in South Asia, which was higher than in other regions.^43^

In this study, susceptibility rates remained mostly stable with a slight increase in levofloxacin susceptibility towards the end of the study period. This stability is corroborated by a study in Europe examining the rates of antimicrobial resistance to *H. pylori* between 2013 and 2020 and a systematic review in the USA between 2011 and 2021.^23,44^ On the other hand, several studies in Asia demonstrated an increase in resistance over time.^43,45^ This was particularly true for levofloxacin, which demonstrated an increase in resistance ranging from 44% in 2013–2014 to 62% in 2017–2018 in South Korea.^46^ This likely developed from the administration of fluoroquinolones for respiratory or urinary tract infections, or due to the emergence of cross-resistance with other fluoroquinolones.^44^

Limitations of this study include the retrospective single-centre design, although this was necessary to generate a large sample size (*n* = 579), ensure AST methods were consistent and allow samples with variable transportation times to be included for analysis. A portion of samples in this study did not have transportation time documented and were excluded from that particular analysis. Our microbiology laboratory did not perform rifabutin or rifampicin susceptibility testing as these agents are not used locally at our centre and rifampicin gradient strips were not available at the time this testing was first implemented. Clinical data (including prior treatment regimens, compliance with therapy and patient outcomes) were not available to the laboratory for this study; however, it is presumed patients undergoing endoscopy for *H. pylori* culture and AST are likely refractory cases, having failed first-line therapy. These patients are therefore not representative of the general population who would typically present to primary care.

### Conclusion

This study highlights factors affecting *H. pylori* culture positivity and the prevalence of antimicrobial resistance in treatment-refractory cases. Although the study analyzed *H. pylori* culture and AST results from a single clinical laboratory covering a wide geographical area within one province, the notably high rate of antimicrobial resistance compared to other centres is concerning. This retrospective review of cumulative susceptibility data showed resistance to multiple antibiotics, including metronidazole and clarithromycin, which are commonly prescribed according to current treatment guidelines. Therefore, for refractory infections, a review of previous antibiotic use and performance of AST to inform tailored treatment regimens is advisable. Our findings could be used to inform local treatment regimen selection for patients with a history of prior treatment failure. Further investigations are warranted to ascertain if our findings are consistent across different regions to provide a clearer understanding of national *H. pylori* antimicrobial resistance patterns.
